# Implementing Translational Research to Understand the Future of COVID-19 and Its Long-Term Consequences: A Degrowth Perspective or the Transformation of a Global Emergency?

**DOI:** 10.3390/biomedicines11010117

**Published:** 2023-01-03

**Authors:** Pasquale Ambrosino, Pasquale Moretta, Anna Lanzillo, Roberto Formisano, Mauro Maniscalco

**Affiliations:** 1 Istituti Clinici Scientifici Maugeri IRCCS, Directorate of Telese Terme Institute, 82037 Telese Terme, Italy; 2 Istituti Clinici Scientifici Maugeri IRCCS, Neuromotor Rehabilitation Unit of Telese Terme Institute, 82037 Telese Terme, Italy; 3 Istituti Clinici Scientifici Maugeri IRCCS, Cardiac Rehabilitation Unit of Telese Terme Institute, 82037 Telese Terme, Italy; 4 Istituti Clinici Scientifici Maugeri IRCCS, Pulmonary Rehabilitation Unit of Telese Terme Institute, 82037 Telese Terme, Italy; 5 Department of Clinical Medicine and Surgery, Federico II University, 80131 Naples, Italy

It has now been three years since the novel severe acute respiratory syndrome coronavirus 2 (SARS-CoV-2) first gave rise to a global health crisis [1] and culminated in the declaration of a pandemic in March 2020. From the outbreak’s earliest phases, it became clear that SARS-CoV-2 infection could manifest in many ways, ranging from a completely asymptomatic condition to a serious illness with fatal complications requiring intensive care [2]. Moreover, the evidence of pulmonary abnormalities, functional limitations, and long-term cardiovascular complications soon manifested in a substantial proportion of COVID-19 survivors [3], suggesting the existence of a post-acute COVID-19 syndrome causing residual disability and increased rehabilitation needs [4,5,6,7]. Importantly, this gave rise to a vaccine race [8] which, thanks to a global effort by the scientific community and governments, made possible the development of national prevention strategies based on mass vaccination protocols [9]. To date, an estimated 13 billion vaccines have been administered worldwide [10]. However, in the meantime, a plethora of new variants with different transmissibility rates and virulence have appeared [11]. Overall, the emergence of less virulent variants and the high immunisation coverage have altered the face of the pandemic, generating a significant easing of national restrictions in many countries while considerably reducing the perception of emergency [12]. However, mounting scientific evidence suggests that great caution is still required, as the vaccine fails to offer complete and persistent immunisation [11], with severe COVID-19 outcomes still possible despite full primary vaccination and initial boosters for patients with underlying health conditions [13]. Moreover, regardless of vaccination status, the risks and 6-month burdens of death, hospitalisation, and incident sequelae clearly increase with the number of reinfections [14]. Overall, it seems clear that the pandemic is not over and the emergency is only changing course, with the possibility of evolution towards a new endemic phase [12]. Therefore, the requirement for high-quality preclinical and translational studies on COVID-19 remains unchanged, aiming to elucidate the mechanisms of infection and reinfection by the new variants, as well as the pathogenesis of acute manifestations and long-term complications.

Several original articles [15,16,17,18,19,20,21,22,23,24,25,26,27] and reviews [28,29,30], as well as case reports [31,32] and meta-analyses [33] were published in the first volume of the Special Issue “COVID-19 and Post-Acute COVID-19 Syndrome: From Pathophysiology to Novel Translational Applications” [34], offering insights into the mechanisms and clinical implications of SARS-CoV-2 infection. The potential role of endothelial dysfunction as a fundamental determinant of most clinical manifestations of COVID-19 and its post-acute phase emerged from an intriguing study evaluating 133 COVID-19 survivors and 133 matched controls [18]. The Authors demonstrated that convalescent COVID-19 patients referred to a rehabilitation setting within 3 months of negative swab testing exhibited significantly lower values of endothelium-dependent flow-mediated dilation (FMD), with a direct association between the severity of endothelial dysfunction and residual pulmonary impairment. Accordingly, Rotoli et al. analysed the cell cultures of human lung microvascular endothelial cells (HLMVEC) and proved that the pro-inflammatory mediators released by spike-activated macrophages during infection may amplify the activation of endothelial cells (ECs) [21]. Two interesting articles have instead analysed the blood alterations induced by the vaccine. Thus, vaccination with the mRNA BNT162b2 vaccine was found to induce a robust cell-mediated immunological memory against spike protein, suggesting that the vaccine-induced T-cell response against SARS-CoV-2 can develop independently from B-cells [24]. On the other hand, Colarusso et al. [27] reported that C5b-9 complement complex, C-reactive protein, and lactate dehydrogenase could reach higher levels in COVID-19 survivors than in vaccinated participants without previous infection. Moreover, COVID-19 participants with residual computed tomography alterations showed higher blood levels of interleukin (IL)-1α and transforming growth factor-β (TGF-β), with lower levels of interferon-γ (IFN-γ), compared to both vaccinated subjects and patients without lung abnormalities. Some interesting review articles were also published in the first volume of the Special Issue. Among them, Karn et al. discussed the potential clinical applications of extracellular vesicle-based therapy for COVID-19 [30], while a meta-analysis of clinical studies provided information on the prevalence of residual manifestations during convalescence [33]. In this regard, Yan et al. also reported on the systemic effects of post-acute COVID-19 syndrome, highlighting the need for exercise-based rehabilitation strategies [28].

Overall, this first Issue provided an interesting but non-exhaustive insight into many molecular and clinical aspects of COVID-19 and its post-acute phase. However, given the urgent need for more translational research on this topic, a second volume of the Special Issue was organised, and some interesting articles have been published so far.

By analysing 319 genomic DNA samples from COVID-19 patients, an interesting association between some genetic variants of innate and adaptive immunity proteins with COVID-19 severity was reported. In particular, the authors identified seven single nucleotide polymorphisms (SNPs) statistically associated with disease risk or a severe course [35]. A total of 25 intensive care unit patients were studied by Berghäll et al. [36] to identify the evolution of blood cell phenotypes and immunological markers in critically ill COVID-19 patients. Thus, compared to healthy controls, COVID-19 participants exhibited a leukocyte profile with predominance for myeloid markers, such as cluster of differentiation (CD) 33, and lower surface markers for lymphocytes, including CD127 (a receptor for IL-7). An aberrant expression of CD158d, CD2 and CD19 in the granulocyte population was also documented, thus suggesting the presence of a dysplastic myeloid lineage beyond peripheral phenotypic changes. Conversely, Ramasamy et al. [37] utilised a hamster model of SARS-CoV-2 infection to provide a comprehensive analysis of the disease course in immunocompetent and immunocompromised hosts. This model provided evidence that infected immunocompromised animals may have a more persistent body weight loss and a more severe airway inflammation with bronchial hyperplasia/metaplasia. In addition, immunocompromised hosts showed inadequate neutralizing antibody response against SARS-CoV-2. Consistent with the hypothesis that a deregulated immune response is responsible for more severe disease, another laboratory research exposed alveolar A549 cells to supernatants from spike-activated macrophages [38]. This caused a massive release of inflammatory mediators, particularly IL-8, whose expression was regulated by the activator protein-1 (AP-1). Other molecular pathways in the alveolar cells involved nuclear factor-kB (NF-kB) in the transcription of interferon gamma-induced protein 10 (IP-10) and the C-C motif chemokine ligand 5 (CCL5), while members of the signal transducers and activators of the transcription (STATs) family of proteins drove the expression of all the other cytokines/chemokines tested. The Authors also documented that the chemokines produced by spike-activated macrophages may be responsible for the loss of the barrier integrity in human alveolar epithelial lentivirus-immortalized cells (hAELVi), confirmed by the increased permeability of the monolayers to mannitol. Therefore, they concluded that the alveolar injury and inflammation in COVID-19 may be the consequence of the complex cross-talk between the alveolar epithelium and activated macrophages. Another intriguing research tested the efficacy and safety of intravenous administration of the monocyte–mesenchymal stem cell coculture secretome in an animal model of a cytokine storm, such as that associated with SARS-CoV-2 infection [39]. In this model, the new biological product showed distinct anti-inflammatory, anti-fibrotic, and regenerative effects, with a good safety profile due to the ratio of pro-inflammatory (e.g., IL-1, IL-6, IL-17, IL-18) and anti-inflammatory cytokines (e.g., IL-10, IL-1RA, insulin growth factor-1) being 0.00042.

Two interesting review articles have also been published in this second volume of the Special Issue. First, the role of the endothelium in the pathogenesis of COVID-19 and its long-term outcomes was studied [40]. The clinical and laboratory evidence of endothelial dysfunction in COVID-19 was summarised, focusing on the direct and indirect mechanisms through which the virus determines the loss of functional and structural integrity of ECs. In particular, the Authors reported the earliest autopsy evidence suggesting a direct cytopathic effect of the virus, also highlighting the role that some inflammatory cytokines (i.e., IL-1, IL-6, and tumor necrosis factor-α) may have in the determination of an increase in the expression of adhesion and prothrombotic molecules on ECs. Given the key pathogenic role of endothelial dysfunction in this clinical setting, the pharmacological and exercise-based rehabilitation strategies targeting endothelial dysfunction were also described by the Authors as attractive therapeutic options. In the systematic review by Gentile et al [41], the potentially significant role of the lung-brain axis in the pathogenesis of respiratory failure in COVID-19 was analysed. Considering how SARS-CoV-2 can target selective neuroanatomical structures, the authors summarized the evidence in the literature that suggested the involvement of this primordial area.

Overall, only a limited fraction of the complex and multiple molecular aspects of COVID-19 have been identified despite the vast number of articles published since the beginning of the pandemic. Interestingly, this number far exceeds that of papers dedicated to other respiratory diseases, including chronic obstructive pulmonary disease, which have been studied for decades. Although only 3 years have passed, the growing scientific evidence on this new clinical condition is expected to increase further, as this reflects the enormous health, social, economic, and political impacts caused by the pandemic worldwide. From our relatively small collections of articles, considerable uncertainty still emerges regarding the molecular mechanisms of SARS-CoV-2 infection and its consequences in various organs and tissues. Moreover, considering the virus’ ability to mutate into new strains, inadequate knowledge exists concerning current variants and those that could appear over time. Therefore, further translational research is needed to better identify the inflammatory pathways during infection with different variants and in vaccinated subjects and to enable new therapeutic strategies. This second volume of the Special Issue, entitled “COVID-19 and Post-Acute COVID-19 Syndrome: From Pathophysiology to Novel Translational Applications (Volume II)”, focuses on the molecular pathways of SARS-CoV-2 infection and its diagnosis, as well as the identification of new prognostic biomarkers and therapeutic strategies targeting endothelial dysfunction, inflammation, and oxidative stress in COVID-19.
